# Stimulant and Atypical Antipsychotic Medications For Children Placed in Foster Homes

**DOI:** 10.1371/journal.pone.0054152

**Published:** 2013-01-09

**Authors:** L. Oriana Linares, Nuria Martinez-Martin, F. Xavier Castellanos

**Affiliations:** 1 Mount Sinai School of Medicine, Adolescent Health Center, New York, New York, United States of America; 2 New York University School of Medicine, Child Study Center, New York, New York, United States of America; 3 Nathan Kline Institute for Psychiatric Research, Orangeburg, New York, United States of America; The University of Queensland, Australia

## Abstract

**Objectives:**

The purpose of this study is to examine the use of prescribed psychoactive medications in a prospective cohort of children shortly after they entered foster homes; and to identify demographics, maltreatment history, psychiatric diagnoses including ADHD comorbidity, and level of aggression that contribute to prescribed use of stimulant and atypical antipsychotic medication over time.

**Methods:**

The sample included N = 252 children (nested in 95 sibling groups) followed for three years up to 4 yearly waves.

**Results:**

Nearly all (89%) met criteria for at least one of eight psychiatric diagnoses and 31% (75/252) used one or more prescribed psychoactive medications. Over half (55%) were diagnosed with Attention Deficit Hyperactivity Disorder (ADHD); of these 38% used stimulants and 36% used atypical antipsychotics. Of the 75 medicated children, 19% received ≥3 different classes of drugs over the course of the study. Stimulants (69%) and atypical antipsychotics (65%) were the most frequently used drugs among medicated children. Adjusted odds ratios (AOR) showed that male gender (AOR = 3.2; 95% CI = 1.5–9.3), African American vs Latino ethnicity (AOR = 5.4; 95% CI = 2.1–14.2), ADHD regardless of Oppositional Defiant (ODD) or Conduct (CD) comorbidity (AOR = 6.0, 95% CI = 1.3–27.5), ODD or CD (AOR = 11.1, 95% CI = 2.1–58.6), and Separation Anxiety (AOR = 2.0, 95% CI = 1.0–4.0) psychiatric disorders were associated with the use of prescribed stimulants; while male gender (AOR = 3.8, 95% CI = 1.5–9.3), African American vs Latino (AOR = 5.1, 95% CI = 1.2–9.2) or Mixed/Other ethnicity (AOR = 3.3, 95% CI = 1.9–13.7), ADHD regardless of ODD or CD comorbidity (AOR = 5.8, 95% CI = 1.2–28.7), ODD or CD (AOR = 13.9, 95% CI = 3.3–58.5), Major Depression/Dysthymia (AOR = 2.8, 95% CI = 1.1–6.7) psychiatric disorders, and history of sexual abuse (AOR = 4.6, 95% CI = 1.3–18.4) were associated with the use of prescribed atypical antipsychotics.

**Conclusion:**

The aggressive use of atypical antipsychotics, which has unknown metabolic risks, suggests that the efficacy and safety of such treatment strategies for psychiatrically ill children in foster care should be monitored.

## Introduction

There were 415,000 children in the United States in foster care in 2010 [1]. Children in foster care experience environmental, social, biological and psychological risks factors prior to and during their stay in care that make them particularly vulnerable to problems of over-activity and inattention [2], high aggression, and high rates of disruptive behavior disorders including Attention Deficit Hyperactivity (ADHD), Oppositional Defiant (ODD), and Conduct Disorders (CD) requiring multilevel treatments including psychiatric interventions [3], [4], [5]. Children placed in foster homes experience higher rates of physical and emotional problems than those in the general population; approximately 60% have a chronic medical condition, and 25% have ≥3 chronic problems; and developmental delays are present in approximately 60% of preschoolers. Children in foster care use both inpatient and outpatient mental health services at a rate 15 to 20 times higher than the general pediatric population. Between 40%–60% are found to meet criteria for at least one psychiatric disorder [6].

Nationwide, the use of prescribed psychotropic medications has increased two to three fold in recent decades for children and adolescents in general and particularly for children served in public sectors. For example, youth in foster care are significantly more likely to be prescribed psychotropic medications than same-age youth in the community [7], [8], [9]. Given the high rates of DSM-IV disorders and their increased access to mental health services after maltreated children enter care, their risk for aggressive psychopharmacology is high [10], [11]. In a national probability sample of 3114 children in the child welfare system, 14% were taking psychotropic medications which is two to three times the rates of children in the community [12]; with considerable geographic variation in medication rates (0%–40%) across localities [13]. Rubin et al. [14] recently reported increased rate of antipsychotic use from 8.9% to 11.8% across 45 states over the period of 2002–2007. Zito et al. [15] examined a sample of 472 children and adolescents in the Texas foster care system that were randomly selected from the 12,189 youth (38% of 32,135 enrollees) who had been dispensed a psychiatric medication according to Medicaid records in the preceding year. They found that 41% of medicated children in foster care were being treated with three of more classes of psychotropic medications. The three most frequent classes were antidepressants, drugs for treating ADHD, and antipsychotics, each of which were used in over 50% of treated youth. Given recent concerns regarding the metabolic adverse effects of treatment with atypical antipsychotics [16]–[18], it is important to understand factors associated with elevated risk for prescriptions for antipsychotics use in this high risk child population.

The purpose of this study is to examine the cross-sectional use of prescribed psychoactive medications among children shortly after they entered foster homes and followed up to 4 yearly waves; and to identify demographic, maltreatment history, psychiatric diagnoses including ADHD comorbidity, and level of aggression that are associated to prescribed use of stimulant and atypical antipsychotic medication classes, in particular. We focused on the use of these psychoactive medication for ADHD and comorbidity with other disruptive behavior disorders (ODD and CD) because they are the most salient disorders among youth in foster care [19], [20]; and because of the increasing concerns about the potential metabolic adverse effects of antipsychotic medications [17]. In addition, we also explored polypharmacy treatment with both stimulant and atypical antipsychotic medication in a small subsample of children.

## Methods

### Sample selection

The sample was drawn from 560 sibling groups referred consecutively from 12 participating foster care agencies in New York City and prospectively followed for four consecutive yearly waves from 2002 to 2007. From referring sibling groups, 19% (104/560) were eligible for enrollment; 2% (9/560) refused. Ineligible sibling groups were excluded because of kinship placement (45%), falling outside the 3–14 age range (27%), imminent discharge (14%), developmental disability (4%), or other (10%).

The prospective cohort consists of children with documented child maltreatment histories of child neglect or abuse as ruled by the local Child Protective Services (CPS) agency, occurring within 6 months of the reporting event. Only siblings removed from their home and placed together in a foster home were included. A sibling was defined as a child who shared a maternal blood tie with, and had the same home environment prior to placement as one or more other children. In addition, enrolled participants met the following criteria: they had no known disabilities, such as a pervasive developmental disorder, sensory deficits or intellectual disability. They were placed in a certified nonkinship foster home, defined as a family-type home where the daily care of a foster child is provided by a nonkinship approved foster parent(s), supervised by a caseworker employed by an authorized agency. Their caregivers were proficient in English and/or Spanish.

Children were participants in a main study examining changes over time in the quality of the sibling relationship following initial foster placement [21], [22]. The inclusion of children who are a part of sibling groups is clinically important in medication studies because the majority (60%) of children placed in foster homes is a part of a sibling group [1]; from a methodological perspective, hierarchical linear modeling procedures permits to statistically control for the effects of data clustering due to sibship.

### Participants

At initial placement (Wave 1) the sample included N = 252 children nested in 95 sibling groups of 2-to-6 siblings (40% were 2-sibs, 35% were 3-sibs, and 25% were ≥4-sibs). The sample was drawn from 560 sibling groups referred consecutively from 12 participating foster care agencies in New York City. From referring sibling groups, 20% (n = 95) were eligible for enrollment; 2% refused. Ineligible sibling groups were excluded because of kinship placement (45%), falling outside the 3–14 age range (27%), imminent discharge (14%), developmental disability (4%), or other (`10%). Children were assessed at four yearly waves shortly after admission to foster homes using a multi-informant (biological parent, foster parent, and teacher) approach. A multi-informant strategy including both caregivers was considered valuable because of family fragmentation and high home instability commonly experienced by children placed in foster homes. Data are based on four assessments: Wave 1 occurred within 4–12 weeks (for 85% of the sample) from initial placement; Wave 2 occurred 12.1±1.5 (M ± SD) months later; Wave 3 occurred 13.0±2.4 months later; and the final assessment (Wave 4) was conducted 10.3±2.5 months later. Four waves starting with initial placement were gathered to capture the typical length of foster stay [1].

Independent assessment of type of child maltreatment gathered from the official CPS records using the Maltreatment Classification System [23] revealed neglect in 79% and abuse in 22% (18% physical abuse and 5% sexual abuse) of cases.

The mean age of biological parents was 32.8 years while that of foster parents was 49.2 years *t* (194) = −13.26, *p*<.001. Parents had a similar number of years of education (10.5 vs. 11.9 years for biological parents and foster parents, respectively). Foster parents were experienced at fostering children (mean 6.8 years), and had an average of 4.5 children in their home.

### Procedure

The protocol was approved by the New York University School of Medicine Institutional Review Board (IRB) and by New York State and local IRBs with legal jurisdiction over the children in the study. An introductory letter was sent to the biological parent (in all cases except two, the biological parent was the mother) and foster parent, with postage-paid postcard included to give the caregiver an opportunity for active refusal. No further contact was made with biological parents who returned postcards within 10 days of mailing (none of the foster parents opted out). Written informed consent was obtained from the biological parents and foster parents. Both parents were interviewed face-to-face in their homes or at the agency in English (83%) or Spanish (17%). The biological parents and foster parents were compensated $50 per visit. Classroom teachers provided measures of child behavioral problems and social competence.

### Measures

Use of prescribed psychoactive medication. Biological and foster parents were interviewed for up to 4 yearly assessments to gather current psychiatric diagnosis and psychoactive medication use. We asked whether children were being administered one or more prescribed medications for ‘*being overactive or having trouble paying attention*’. To reduce recall bias, we asked only about current medications. For parents who reported use, we inquired for the trade name for each drug. If the name was not recalled, the parent was encouraged to show the medication container (trade names for 10 children's medications remained unknown). Drugs were coded into seven non-overlapping classes: psychostimulants, non-psychostimulants for ADHD (atomoxetine, guanfacine, clonidine), atypical antipsychotics, typical antipsychotics, antidepressants and antianxiety agents, mood stabilizers, and other, based on the Children's Medication Chart [24] supplemented by the Physicians Desk Reference [25].

### Explanatory variables

#### Gender and age categories

At the outset of the study, children ranged in age from 3 to 14 years of age. For logistic regressions, they were grouped into three age ranges: 3.0–7.9, 8.0–11.9, and ≥12 years of age.

#### Ethnicity

Children were grouped into three main ethnic categories: African American (AA) or African descent children (n = 118), Latino (n = 66 out of which 40 were Puerto Rican) and Mixed Ethnicity/Other (n = 68). The Mixed Ethnicity/Other group included 51children who were of mixed minority ethnicity (69% of them were Latino and AA), and 17 children who were of Caucasian or Asian background.

#### Type of child maltreatment

Based on the Maltreatment Classification System (MCS) [23], children were classified as suffering from neglect (failure to provide, lack of supervision; or emotional, medical, educational, or legal neglect), exposure to domestic violence, and abuse (physical abuse or sexual abuse). Trained coders blind to participant history independently recoded 10% (25/252) of randomly selected cases, yielding kappa coefficients of .89 and .82 for aggregated neglect (including exposure to domestic violence) and abuse types, respectively.

#### Psychiatric diagnoses

Diagnosis were determined using published age-dependent SAS algorithms for the Computerized Diagnostic Interview Schedule for Children-4 (C-DISC4) Parent Version (Generic English, Generic Spanish and Experimental Young Child) [26], [27]. Eight C-DISC4 modules were administered by trained research assistants. The modules are: Attention Deficit Hyperactivity Disorder (ADHD), Oppositional Defiant Disorder (ODD), Conduct Disorder (CD), Separation Anxiety Disorder (SAD), Generalized Anxiety Disorder (GAD), Posttraumatic Stress Disorder (PTSD), Major Depression/Dysthymia (MDD), and Elimination Disorders (Encopresis and Enuresis). The age of onset criteria were not considered in diagnostic determination because foster parents had limited information about this criterion and prior cohabitation of children with their biological parent was frequently interrupted. We combined informant data so that a diagnosis was considered present if it was endorsed by either informant at any of the four waves [28], [29].

#### ADHD comorbidity

Children were classified across waves (1–4) as: undiagnosed ADHD, ADHD+/− =  diagnosed ADHD regardless of ODD or CD comorbidity, ADHD−  =  ADHD no comorbidity, and ADHD+ =  ODD or CD comorbidity.

#### Behavior problem scales

The Eyberg Child Behavior Inventory-Parent Report (ECBI-PR) [30], a dimensional measure of externalizing problems for ages 2–17, was gathered from parents to obtain the ECBI-PR Total Problems and Aggression. The ECBI-PR Total measure consists of 36 items which yield an intensity problem score; it has been shown to correlate with independent observations of children's behavior and to differentiate clinic-referred and non-clinical populations [30]. The Cronbach alpha coefficient for ECBI-PR Total was .95. The Aggression subscale of the ECBI-PR consists of 6 items (*hits parents; destroys toys or other objects; verbally fights with friends own age; verbally fights with sisters and brothers; physically fights with friends own age; physically fights with sisters and brothers*) which showed high internal consistency (alphas averaging .82) and adequate item factor loadings (averaging .74). Children who scored z≥1.5 SD above the sample mean were classified as Aggression+. The Sutter Eyberg Student Behavior Inventory-Revised (SESBI-R Total) [30] is a 38-item teacher counterpart to the ECBI-PR to assess conduct problems in the classroom. The Cronbach alpha coefficient for SESBI-R Total was .95.

### Data analyses

The use of psychoactive medications was dichotomized (Y/N) as: (1) stimulants and (2) atypical antipsychotics, gathered from parental report of prescribed psychoactive medication across any of the 4 data collection waves. We also described the group of polypharmacy users of stimulants and atypical antipsychotics, but their small number precluded further analyses. We examined the associations among medications, demographics (gender, age category, ethnicity), and study variables [C-DISC4 diagnosis, ECBI-PR Total Problems and Aggression+ (parent), SESBI-R Total Problems (teacher) and history of maltreatment] using logistic regression models in the context of generalized estimating equations [31]. The GEE approach (implemented in SPSS V 19.0) adjusted for the clustered observations (children in families and repeated measurements). In addition to the estimated odds ratios (OR), we report 95% confidence intervals (95% CI) and *p* values based on robust standard errors from the GEE analyses. We proceeded in two steps: first, we examined bivariate (unadjusted) associations between medication prescriptions and a range of demographic and clinical variables. Although this step was explicitly exploratory, we only retained variables that were associated at the .01 alpha level or less for the multivariate analysis. Next, we entered all of the variables with bivariate associations into multivariate GEE models using adjusted OR to examine their unique contribution of study variables on prescribed use of stimulants or atypical antipsychotics.

## Results

Table 1 shows psychosocial characteristics, C-DISC4 diagnoses, and use of prescribed psychoactive medication. Of the 252 children in our sample, there was a slight predominance of males (56%); racial and ethnic minorities were highly represented (African-American, 46%; Latinos, 26%); and about half (57%) of the children were in the youngest age group (3.0–7.9 years old). Over 76% of the children were classified as neglected, including a substantial proportion (40%) who had been exposed to domestic violence; and 22% were classified as abused, with physical abuse about four times as frequent as sexual abuse. At Wave 1, 35% and 30% of the children were at the clinical range for ECBI-PR Total and SESBI-R Total (T score ≥60) respectively.

**Table 1:** Child psychosocial characteristics, psychiatric diagnoses, and prescribed use of psychoactive medications across the three-year study period.

N Total	252%
Gender: Boys N (%)	141 (56)
Age M (SD)	7.76 (3)
Age category (yrs) N (%)	
Ages 3.0–7.9	143 (57)
Ages 8.0–11.9	85 (34)
Ages ≥12	24 (9)
Ethnicity N (%)	
African American	118 (46)
Latino	66 (26)
Mixed/Other (Caucasian, Asian)	68 (27)
Type of child maltreatment (MCS) N (%)^a^	
Neglect	90 (36)
Exposure to domestic violence	99 (40)
Physical abuse	45 (18)
Sexual abuse	11 (5)
Psychiatric diagnoses (C-DISC4) N (%)	
ADHD +/−b	138 (55)
ADHD – (no ODD or CD)	50 (20)
ADHD + (comorbid with ODD or CD)	88 (35)
Oppositional Defiant (ODD)	100 (40)
Conduct (CD)	63 (25)
Separation Anxiety (SAD)	117 (46)
Generalized Anxiety (GAD)	20 (8)
Posttraumatic Stress (PTSD)	15 (6)
Major Depression (MDD)	29 (12)
Elimination	54 (21)
Psychoactive medication use N (%)	
Stimulants	52 (69)
Non-stimulants for ADHD	19 (25)
Atypical antipsychotics	49 (65)
Typical antipsychotics	1 (0)
Antidepressant/Anxiolytics	5 (7)
Mood stabilizers	10 (13)
Other	3 (4)
Behavior problem scales	
ECBI-PR Total (T≥60)	88 (35)
ECBI-PR Aggression+ (>1.5 SD)	44 (10)
SESBI-R Total (T ≥60)	76 (30)

a Maltreatment Classification System. Five cases were excluded due to missing data. Percentages do not sum to 100% due to multiple types of maltreatment. ADHD  =  Attention Deficit Hyperactivity Disorder.

b ADHD +/−  =  ADHD regardless of ODD or CD comorbidity.

Across subtypes, ADHD+/− diagnosis (regardless of comorbidity with ODD or CD), was reported for a majority of the sample (55%), followed by SAD, ODD, CD, Elimination Disorder, MDD, GAD, and PTSD. Nearly one quarter (23%) met criteria for at least two different diagnoses, 20% for three, 14% for four, and 9% had five or more diagnoses.

There were 31% (75/252) children who were reported by their caregivers to have used psychoactive medication for symptoms of overactivity or difficulty paying attention: 22 children used stimulants only, 19 used atypical antipsychotics only, 30 used both, and 4 used other medication. These 4 medicated children were excluded from further analyses because they were treated with non-stimulants for ADHD (n = 2) and mood stabilizers (n = 2) resulting in a sample size of N = 248 for the subsequent analyses. As Table 1 shows, 52 children were treated with stimulants, 49 with atypical antipsychotics, and 30 used both. These two classes were the most commonly used medications; each used by about two-thirds of the medicated children. Non-stimulant medications for ADHD were used by one quarter of medicated children. Use of mood stabilizers, serotonin selective reuptake inhibitors, and others was reported less frequently. There were 35 children who used one medication, 26 who used two different medications and 14 who used three or more different medications.

As seen in Table 2, as compared to the undiagnosed ADHD children, the 137 children diagnosed with ADHD+/− were more likely to use stimulants (30% vs. 10%, χ^2^ = 13.50, *p*<.001), atypical antipsychotics (28% vs. 10%, χ^2^ = 12.29; *p*<.001) and both stimulants and atypical antipsychotics (18% vs. 5%, χ^2^ = 13.50; *p*<.001). As compared to ADHD− children, the 88 children diagnosed with ADHD+ were significantly more likely to use stimulants (38% vs. 16%, χ^2^ = 23.06; *p*<.001), atypical antipsychotics (38% vs. 10%, χ^2^ = 27.08; *p*<.001) and both (25% vs. 6%, χ^2^ = 23.06, *p*<.001). Psychoactive medication use for ADHD− children did not differ from undiagnosed ADHD children. Compared with the 36 children classified with Aggression-, the 44 children classified with Aggression+ (ECBI-PR subscale), 16% used stimulants (41% vs. 18%, χ^2^ = 10.88, *p* = .001), 39% atypical antipsychotics (39% vs. 16%, χ^2^ = 12.02; *p* = .001), and 25% both stimulants and atypical antipsychotics (25% vs. 10%, χ^2^ = 6.34; *p* = .012).

**Table 2:** Rates of prescribed use of target psychoactive medications by psychiatric diagnosis (C-DISC4) and level of aggression (n = 248)^a^.

		Prescribed Use of Psychoactive Medication Classes					
C-DISC4 psychiatric diagnoses		Stimulants		Atypical antipsychotics		Stimulants and atypical antipsychotics	
	N	N = 52	69%	N = 49	65%	N = 30	44%
Undiagnosed ADHD	114	11	10%	11	10%	5	5%
ADHD +/−^b^	137	41	30%	38	28%	25	18%
			p<.001		p = .001		p<.001
ADHD− (no ODD or CD)	49	8	16%	5	10%	3	6%
ADHD+ (comorbid with ODD or CD)	88	33	38%	33	38%	22	25%
			p<.001		p<.001		p<.001
ECBI-PR Aggression −	204	36	18%	32	16%	22	10%
ECBI-PR Aggression +	44	18	41%	17	39%	11	25%
			p = .001		p = .001		p = .012

Note: ADHD  =  Attention Deficit Hyperactivity Disorder; ODD = Oppositional Defiant Disorder; CD = Conduct Disorder.

a Excludes 4 children who used other than the target study medication classes. ^b^ADHD −/+ =  ADHD regardless of comorbidity with ODD or CD.

Table 3 shows bivariate odds ratios (OR) associated with explanatory variables (demographics, C-DISC4 diagnosis, total problems, aggression level, and history of child maltreatment) for prescribed use of stimulants and atypical antipsychotics as well as OR estimates from the multivariate models. In the bivariate GEE analyses, greater use of stimulant medications was associated with male gender and African American (vs Latino) ethnicity, ADHD+, ADHD −/+, ODD, CD, SAD, and ECBI-PR Aggression+. Greater use of atypical antipsychotic medications was associated with male gender, African American (vs Latino and Mixed/Other ethnicity), ADHD −/+, ODD, CD, MDD, and history of sexual abuse. Four of the eight C-DISC4 disorders were associated with use of stimulants and atypical antipsychotics; SAD was associated with stimulants but not atypical antipsychotics, while MDD was associated with atypical antipsychotics and not stimulants. GAD, PTSD and Elimination Disorders were not associated with either.

**Table 3:** Unadjusted and adjusted associations between study variables and target prescribed psychoactive medications^a^.

Study variables	Stimulants		Aypical Antipsychotics	
	Bivariate	Multivariate	Bivariate	Multivariate
	OR(95% CI)*		OR(95% CI)	
**Demographics**				
Gender (boy)	3.61(1.92–6.77)***	3.22(1.61–6.47)***	3. 3. 09(1.53–6.20)**	3.75(1.52–9.25)**
Age (≥12 vs. 3.0–7.9)	1.97(.66–5.90)		1.61(.56–4.60)	
Ethnicity				
AA vs Latino	4.82(1.97–11.80)***	5.35(2.01–14.20)***	3.39(1.34–8.59)**	5.10 (1.15–9.19)***
AA vs Mixed/Other	2.07(.80–5.37)		3.72(1.54–9.02)**	3.26 (1.9–13.67)*
**C-DISC4 psychiatric diagnoses**				
ADHD–(without ODD or CD)	1.38(.60–3.14)		2.17(1.05–4.47)	
ADHD + (with ODD or CD)	4.12(2.10–8.08)***	.24(.04–1.55)	5.02(2.81–8.98)***	.563(.22–1.43)
ADHD+/−	3.80(1.89–7.63)***	5.99(1.31–27.51)*	3.51(1.80–6.87)***	5.83(1.18–28.73)*
ODD	4.72(2.45–9.11)***	11.09(2.10–58.62)**	4.59(2.54–8.30)***	13.91(3.31–58.49)***
CD	2.89(1.61–5.19)***		2.81(1.51–5.21)***	
SAD	2.29(1.28–4.12)**	2.00(.99–4.03)*	1.70(.86–3.37)	
GAD	3.05(1.15–8.12)		3.19(1.21–8.43)	
PTSD	1.75(.68–4.53)		1.22(.39–3.88)	
MDD	2.76(1.25–6.12)		3.17(1.35–7.42)**	2.76(1.14–6.69)*
Elimination disorder	.62(.29–1.31)		1.06(.47–2.43)	
**Behavior scales**				
ECBI-PR Total	2.16(.61–7.69)		4.77(1.36–16.8)	
ECBI-PR Aggression+	2.88(1.36–6.12)**	1.65(.72–3.77)	3.42(1.49–7.86)	
SESBI-R Total	.69(0.19–2.48)		2.57(.91–7.31)	
**History of maltreatment**				
Physical abuse	.90(.33–2.45)		1.79(.72–4.42)	
Sexual abuse	2.40(.67–8.54)		4.50(1.58–12.82)**	4.56(1.32–18.40)*
Exposed to DV	1.57(.71–3.44)		1.73(.82–3.62)	

Note. ^a^ Odds ratios (OR), 95% confidence intervals (CI) are shown for predictor variables for prescribed use of stimulants and atypical antipsychotics. The estimates were derived by bivariate (unadjusted) and multivariate (adjusted for study variables) generalized estimating equation models.

* = *p*<.05; ** = *p*<.01; *** = *p*<.001.

AA  =  African American; ADHD −/+ =  Attention Deficit/Hyperactivity regardless of ODD or CD; ODD  =  Oppositional Defiant; CD  =  Conduct; SAD  =  Separation Anxiety; GAD  =  Generalized Anxiety; PTSD =  Posttraumatic Stress; MDD  = Major Depression/Dysthymia. DV =  domestic violence.

After adjusting for the other explanatory variables, adjusted odds ratios (AOR) in the multivariate GEE models show that children on stimulants were more likely to be male (AOR  = 3.26), of African American ethnicity vs Latino (AOR  = 5.35), have ADHD+/− (AOR = 5.99), ODD or CD (entered together due to multicollinearity; AOR  = 11.09, and SAD AOR  = 2.00). Children on atypical antipsychotics were more likely to be male (AOR  = 3.75), African American vs Latino (AOR  = 5.10) or Mixed/Other (AOR  = 3.26), have ADHD+/− (AOR  = 5.99), ODD or CD (AOR = 13.91), and MDD (AOR  = 2.76) and history of sexual abuse (OR = 4.56). None of the other C-DISC4 diagnoses remained significant in these multivariate models. Goodness of fit indices for the above GEE models were: QICC = 206.570 for stimulants and QICC = 255.522 for antipsychotics.

We also examined the smaller subsets of children (not shown) who used stimulants only (n = 22), atypical antipsychotics only (n = 19), and both stimulants and atypical antipsychotics (n = 30). Multivariate analysis showed that ODD or CD diagnosis was associated with use of stimulants alone [OR  = 10.62; 95% CI: 1.21–3.39; *p* = .03]. While prescribed use of both stimulants and atypical antipsychotics was significantly associated with C-DISC4 diagnoses (except ADHD−, SAD, PTSD, and Elimination Disorders), the highest risk was associated with ODD diagnosis [OR = 4.64; 95% CI: 2.42–8.91; *p*<.001]; on the multivariate analysis, however, none of the diagnoses remained significant in the model. Instead, one type of child maltreatment (exposure to domestic violence) was found as a significant contributor in the model [OR = 2.69; 95% CI 1.18–6.13; p = .019].

## Discussion

As shown in other cross sectional studies [32], [10], [33], our sample of children in foster care in New York City exhibited a particularly high prevalence of Attention Deficit/Hyperactivity (55%), Oppositional Defiant (40%), and Conduct (25%) disorders. The high rates of Separation Anxiety disorder (46%) shown in our recent entrants to foster care are important to highlight; this finding may reflect the young age (mean of 7.76) of the children enrolled in the study and our multi-source approach in which the biological parent also contributed to psychiatric diagnostic data.

Use of prescribed atypical antipsychotics was found to be almost identical to that of stimulants (65% vs 69% among medicated children) suggesting aggressive clinical treatment with antipsychotics. One of the most notable recent trends in psychopharmacology has been the increased use of atypical antipsychotics across the lifespan and among youth [34]–[36]. Based on 3,466 psychotropic visits to office-based physicians between 1996–2007, Comer et al., [36] recently found that while there has not been an increase of child comorbid disorders, the co-prescription of ADHD and antipsychotic medications has increased from 14% to 20% among office-based practices from 1996–2007 (AOR  = 6.22, 95% CI  = 2.82–13.70, *p*<.0001). Our data is consistent with the recent rise of polyclass medication use of two psychotropic classes [37], [14] although not necessarily used concomitantly in the children in this study. Disruptive behavior disorders, primarily including CD and ODD in children and adolescents are associated with aggression and poor short and long-term outcomes. Although no pharmacotherapy for these conditions is currently approved for pediatric use, evidence suggests that atypical antipsychotic treatment may be useful in children with these conditions who present with problematic aggression in which impulsive/reactive aggression may be a key treatment target [38]. Potential pharmacotherapy for CD with marked aggression includes mood stabilizers, typical antipsychotics and atypical antipsychotics [39].

Having focused on psychoactive medication use for child overactivity and inattention problems, univariate results showed that about 30% (41/137) of children who met C-DISC4 criteria for ADHD at any point over the course of this three-year study were reported to be on stimulant treatment. By comparison, Jensen et al. [40] found that only 12.5% of children in the community meeting criteria for ADHD had been prescribed stimulants in the prior year. More recent community studies have found stimulant treatment rates in children meeting criteria for ADHD as low as 32% [41], 59% of male and 46% of female twins [42], reaching up to nearly 75% over a four-year period in the Great Smoky Mountain Study [43]. Thus, the 30% rate of reported stimulant use in our sample of children in foster care falls within the range of other non-foster care samples.

In a hard-to-track sample of maltreated children entering foster homes, this prospective cohort study of psychoactive medication use over a three year period uses a multi-source approach for data collection including: maltreatment history, yearly structured psychiatric diagnoses, and behavior assessments in the foster home and the classroom. We found a remarkably comparable medication rates for the use of stimulants and atypical antipsychotics in this sample. Multivariate data showed ADHD+/− regardless of ODD or CD diagnoses (AOR = 5.99 and AOR = 5.83), and ODD or CD (AOR = 11.09 and AOR = 13.91) was associated with stimulants use and with atypical antipsychotics, respectively. The associations between these research-gathered diagnoses of disruptive behavior disorders and prescribed stimulants and antipsychotics suggest that cardinal symptoms of ADHD singly or in combination with ODD or CD phenotypes are salient in medication evaluations and treatment by community-based medical providers serving foster children. This usage pattern is consistent with the slight though growing evidence base that atypical antipsychotics may be efficacious for ADHD comorbid children and adolescents [44], [45], [46]–[47]. Still, the potential short-term benefits of atypical antipsychotics for the treatment of such comorbidity must be balanced against the increasingly recognized risks of long-term metabolic derangements including obesity and the metabolic syndrome [16], [17], [48], [18]. More research is needed to identify the long-term efficacy and safety of atypical antipsychotic administration for ADHD comorbid children and adolescents, particularly those who are exposed to the severity of environmental stressors that characterize children in foster care. A history of sexual abuse may be a red flag for increased risk for atypical antipsychotics.

The link in the multivariate analyses between research-based diagnosed Separation Anxiety (SAD) and Major Depression (MDD) and clinician-based stimulants and atypical antipsychotic medications, respectively, points to the need to clinically differentiate behavioral phenotype related to the internalizing disorders among foster children to avoid the risk for misaligning diagnosis and use of psychoactive medication treatment. Children with internalizing problems who receive these study medication classes may present masked externalizing profiles needing further differential diagnosis. Post hoc analyses showed that from the 61 children who were SAD comorbid with ODD or CD and the 20 children who were MDD comorbid with ODD or CD children a larger number received stimulants (43% vs 14%; χ^2^ = 22.89, p<.001) and atypical antipsychotics (55% vs 18%, χ^2^ = 15.205 p<.001) as compared to noncomorbid children, suggesting that careful evaluation and assessment of internalizing disorders may reduce demand for medication use with stimulants or antipsychotics.

Demographics of male gender and ethnic minority status (i.e., African American) were associated with higher rates across the two psychoactive medication classes examined in this study. The effects of gender were fully expected, especially given the focus on aggression and externalizing disorders. By contrast, we were surprised to find a medication disparity between African American children and the other minority groups i.e., Latino children and the Mixed/Other children (mostly of whom were mixed Latino and AA); prior reports show that minority versus white children have decreased rates of use of psychotropic medication but no differences are known within children belonging to ethnic minority backgrounds [49]. This result must be considered provisional since we cannot determine the potential effects of confounding factors such as variations in practice patterns of physicians [50].

These findings cannot be interpreted without considering the limitations of this study. The study is a non-population based descriptive analysis on a strictly defined sample. The study was limited to children who were a part of sibling groups. However, sibships represent a substantial proportion of children in foster care and we accounted statistically for family clustering. Nevertheless, it is possible that physicians may have been influenced by shared family-level factors in unknown ways at the time of their phenotype assessments and choice of medication treatment. The children were placed together as a sibling unit in non-kinship care, so findings may not generalize to children who entered care alone, or those who are placed with relatives in kinship homes.

We limited our inquiry into the psychotropic treatment of children in foster care to the specific indications of ‘*overactivity and trouble paying attention*’. It is unknown how parents understood the probe, the reason/s their child is taking medication, and how parents call the underlying problem, all which may have resulted in underreporting psychoactive medications. The study narrow focus may explain our lower rates of prescription reports of antidepressants/anxiolytics (chiefly serotonin reuptake inhibitors) and mood stabilizers as compared to those from other samples [51], [10]. While reports of medication use were verified by visual inspection of prescription labels, we did not obtain dosages or confirm the degree of adherence with prescribed treatment.

It is also true that we did not have access to agency clinical records or medical diagnoses; however, our diagnostic classifications were systematically obtained from both biological and foster parents over multiple waves and were likely more comprehensive than standard clinical diagnoses [52]–[56].

We cannot address the appropriateness and benefits of individual medication regimens, nor the extent of potential adverse effects. These consequences should be monitored prospectively in all children and adolescents undergoing treatment with atypical antipsychotics, and particularly in vulnerable youth who are entrusted into the foster care system (Correll, 2008a). Children entering the foster care system are a vulnerable population at high risk for externalizing and internalizing psychiatric disorders. The use of atypical antipsychotics to treat disruptive behavior disorders suggest that the efficacy and safety of such treatment strategies for psychiatrically ill children in foster care should be monitored.
